# Controllable synthesis of flake-like Al-doped ZnO nanostructures and its application in inverted organic solar cells

**DOI:** 10.1186/1556-276X-6-546

**Published:** 2011-10-04

**Authors:** Xi Fan, Guojia Fang, Shishang Guo, Nishuang Liu, Huimin Gao, Pingli Qin, Songzhan Li, Hao Long, Qiao Zheng, Xingzhong Zhao

**Affiliations:** 1 Key Laboratory of Artificial Micro- and Nano-structures of Ministry of Education, Department of Electronic Science and Technology, School of Physics and Technology, Wuhan University, Wuhan, 430072, People's Republic of China

**Keywords:** Al-doped ZnO, inverted organic solar cell, electrical conductivity, morphology

## Abstract

Flake-like Al-doped ZnO (AZO) nanostructures including dense AZO nanorods were obtained via a low-temperature (100°C) hydrothermal process. By doping and varying Al concentrations, the electrical conductivity (*σ*) and morphology of the AZO nanostructures can be readily controlled. The effect of *σ *and morphology of the AZO nanostructures on the performance of the inverted organic solar cells (IOSCs) was studied. It presents that the optimized power conversion efficiency of the AZO-based IOSCs is improved by approximately 58.7% compared with that of un-doped ZnO-based IOSCs. This is attributed to that the flake-like AZO nanostructures of high σ and tunable morphology not only provide a high-conduction pathway to facilitate electron transport but also lead to a large interfacial area for exciton dissociation and charge collection by electrodes.

## Introduction

In the recent years, much attention in development of inverted organic solar cells (IOSCs) has been focused on zinc oxide (ZnO) nanostructures as an electron transport layer (ETL), which is attributed to its excellent chemical and thermal stability, high electron mobility, and easy fabrication [1-3]. One of the most popular structures is indium tin oxide/nanostructured ZnO/active layer/molybdenum oxide (MoO_3_)/anode [1,2,4,5]. In such IOSC devices, however, ZnO nanostructures still remain challenging. On the one hand, the electrical conductivity (*σ*) of ZnO nanostructures, which determines the characteristic of electron transfer, is still not high enough for IOSCs. For example, the short-circuit current density (*J*_SC_) of ZnO nanorod (NR)-based IOSCs was usually limited to a small range of 6.0 to 6.5 mA cm^-2 ^under simulated 100 mW cm^-2 ^(AM 1.5 G) solar irradiation [4], which blocks its practical application. On the other hand, the non-absorbing ZnO NR arrays, synthesized via hydrothermal method, are usually too densely packed, making for an insufficient polymer filling fraction, low photon absorption efficiency, and exciton dissociation into free carriers at a donor-acceptor site before recombining [1]. Moreover, owing to the smaller interfacial area between the ZnO NRs and the active layer, the power conversion efficiency (PCE) of ZnO NR-based IOSCs is often lower than that based on nanoparticles [6]. If disperse and ultrathin ZnO nanostructures can be achieved, it will facilitate electron transfer of IOSCs by increasing the interfacial area between ZnO nanostructures and the active layer [4].

Recently, many efforts had been also made to improve the *σ *of ZnO nanostructures by doping various chemical elements such as gallium (Ga) [4,7,8], indium [9], and aluminum (Al) [10,11] into ZnO nanostructures. Among them, Al-doped ZnO (AZO) nanostructures are capable of reaching the highest *σ *without deterioration in optical transmission [11]. Moreover, it had been reported that the *J*_SC _could be improved dramatically by a Ga-doped ZnO NRs, which can increase the *σ *of the ETL and decrease the series resistance (*R*_S_) of IOSCs. Hence, it is possible to increase the device performance by doping Al concentrations in ZnO nanostructures.

In this paper, we demonstrate the performance enhancement of the IOSCs based on the flake-like AZO nanostructures acting as an ETL and a possible electron acceptor. By doping and varying Al concentrations in a solution of 0.025 M zinc nitrate [Zn(NO_3_)_2_·6H_2_O] and hexamethylenetetramine (HMT), we obtained ultrathin flake-like AZO nanostructures of high *σ *and distinct morphology. Moreover, the morphology of flake-like AZO nanostructures, such as the density and the size, can occur to change with varying the Al concentrations. Compared with that of the un-doped ZnO NR-based IOSCs, the PCE of the optimized AZO-based IOSCs has a dramatic increase from 1.04% to 1.65%.

## Experimental section

**Figure 1:**
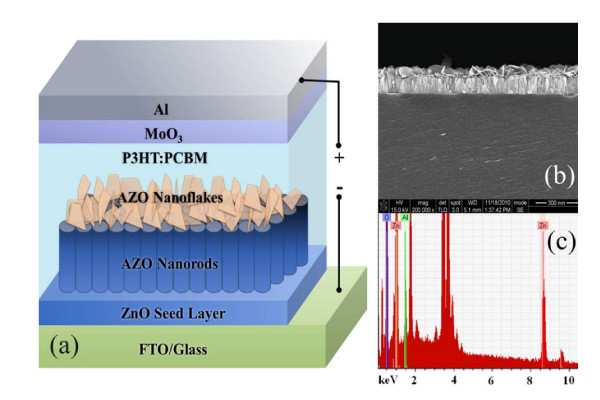
**Schematic structure of the IOSCs, and FE-SEM images and EDS spectrum of AZO nanostructures**. (**a**) The schematic structure of the IOSCs based on AZO nanostructures. (**b**) The cross-sectional FE-SEM images of AZO nanostructure arrays fabricated on FTO substrates in an Al concentration of 4 mM solution. Scale bar, 300 nm. (**c**) The EDS spectrum of AZO nanostructure array sample fabricated on the FTO substrate in an Al concentration of 4 mM solution.

Figure 1a shows the schematic structure of the IOSCs. In the fabrication of the IOSCs, firstly, a 50-nm ZnO seed layer was deposited on fluorinated tin oxide (FTO) substrate at a deposition temperature of 100°C by radio frequency magnetron sputtering from a ZnO target. To prepare flake-like AZO nanostructures, different amounts of aluminum nitrate nonahydrate (Al(NO_3_)_3_·9H_2_O, 99.997%, Sigma-Aldrich, St. Louis, MO, USA) were dissolved in a solution as an Al source to fix its concentration at 1, 2, and 4 mM, respectively. The solution for AZO nanostructure growth was an aqueous solution of 0.025 M Zn(NO_3_)_2_·6H_2_O and HMT. The reaction was kept at 100°C for 1 h, and the ZnO nanoflakes (NFs) exhibited a length of approximately 100 nm, as shown in Figure 1b. Then, the fabricated samples were removed from the solution, rinsed with distilled water, and dried in air. The solution of poly(3-hexylthiophene)/(6,6)-phenyl C_61 _butyric acid methyl ester (P3HT/PCBM, 20:18 mg) in chlorobenzene (1.0 ml) was deposited by spin coating at 1,000 rpm on the AZO NF arrays. Afterwards, MoO_3 _thin film of 10 nm thickness and Al electrodes of 100 nm thickness were deposited on P3HT/PCBM active layer via thermal evaporation at the pressure of 10^-6 ^Torr. The thickness was measured using the thickness monitor (Maxtek, Inc. USA). Finally, the samples were annealed at 150°C for 8 min under argon atmosphere (< 1 ppm O_2 _and < 1 ppm H_2_O). The active area of the IOSCs is 0.2 cm^2^. Figure 1c shows the energy dispersive spectrum (EDS) of AZO nanostructures grown in a solution with 4 mM Al doping, which indicates the effective incorporation of Al atoms into ZnO lattices.

## Results and discussion

**Figure 2:**
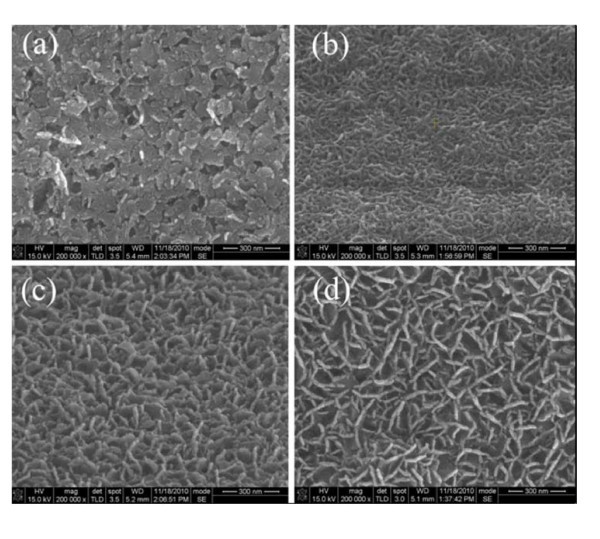
**The SEM images of ZnO nanostructures grown in a solution with different Al concentrations**. (**a**) 0 mM; (**b**) 1 mM; (**c**) 2 mM; (**d**) 4 mM. Scale bar, 300 nm.

Figure 2a, b, c, d shows the field-emission scanning electron microscopy (FE-SEM) images for un-doped and Al-doped ZnO nanostructures grown in a 0.025 M Zn(NO_3_)_2_·6H_2_O and HMT solution with different Al concentrations from 1 to 4 mM. It is observed that the densely packed ZnO NR arrays have grown almost on ZnO seed layer without Al doping. Meaningfully, after the Al concentrations are added in the solution, it is clearly found that disperse and ultrathin flake-like AZO nanostructures have grown. With increasing Al concentrations from 1 to 4 mM, the density of the AZO NFs decreases and the average size of the AZO NFs is 12.5, 13.6, and 15.0 nm, respectively, indicating that the smaller interfacial area between flake-like nanostructures and the active layer is achieved with increasing Al concentrations from 1 to 4 mM. It is shown by EDS that the atomic concentration of Al is 0.7%, 1.25%, and 1.47% corresponding to 1, 2, and 4 mM Al doping, respectively.

**Figure 3:**
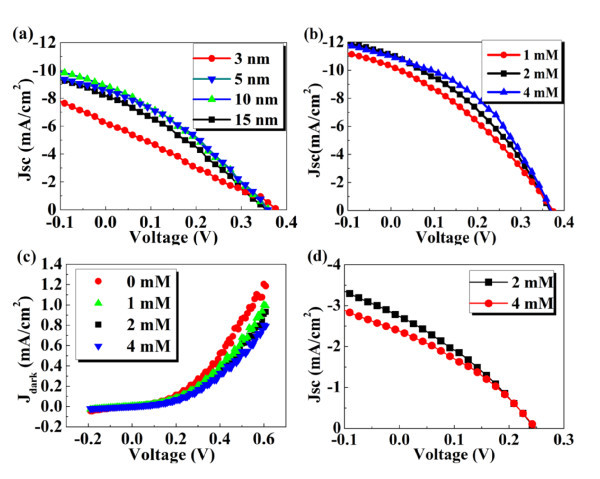
**The *J-V *characteristics and the corresponding dark *J*-*V *characteristics**. (**a**) The *J-V *characteristics of the IOSCs with different MoO_3 _thickness from 3 to 15 nm under simulated 100 mW cm^-2 ^(AM 1.5 G) solar irradiation. (**b**) The *J-V *characteristics of IOSCs with Al concentrations from 1 to 4 mM. (**c**) The corresponding dark *J-V *characteristics of IOSCs with different Al concentrations. (**d**) The *J-V *characteristics of devices based on ZnO/P3HT hybrid solar cells.

To increase the PCE of the IOSC devices, we firstly optimize the MoO_3 _thickness from 3 to 15 nm. Figure 3a shows the current-voltage (*J*-*V*) characteristics of the IOSCs with different MoO_3 _thicknesses from 3 to 15 nm, under simulated 100 mW cm^-2 ^(AM 1.5 G) solar irradiation. It is illuminated that the PCE of the IOSC devices reaches a maximum value of approximately 1.04% with 5 nm thickness of MoO_3_, which is attributed to a relative complete coverage of the active layer by MoO_3_, and thus avoiding certain leakage currents in some weak spots [12]. As increasing the thickness from 10 to 15 nm, the *J*_SC _decreases from 8.87 to 8.18 mA cm^-2 ^and the fill factor (FF) decreases from 0.315 to 0.307, and as a result, the PCE decreases from 1.01% to 0.90%. Previous works had reported that MoO_3 _has a quite high electrical resistivity (**>**10^9 ^Ω cm) [13-15]. Therefore, the deterioration of the IOSC performance may be induced by a higher intrinsic resistance of the MoO_3 _with 15 nm, restraining the charge transport from the active layer to Al electrodes.

Figure 3b shows the *J*-*V *characteristics of the IOSC devices with MoO_3 _of 5 nm thickness and with flake-like AZO nanostructures grown in a solution with Al concentrations from 1 to 4 mM. The performance parameters of the devices are also summarized in Table 1.

**Table 1:** Device performance parameters of the IOSCs and the corresponding electrical conductivity

Sample	*J*_SC _(mA cm^-2^)	*V*_OC _(V)	FF	PCE (%)	*R*_S _(Ω cm^2^)	*σ *(Ω cm)^-1^	Size (nm)
0 mM	8.56	0.360	0.337	1.04	31.5	8.2 × 10^-5^	-
1 mM	10.26	0.375	0.328	1.26	22.7	3.2 × 10^-4^	12.5
2 mM	11.08	0.370	0.351	1.44	17.8	4.4 × 10^-4^	13.6
4 mM	10.97	0.365	0.412	1.65	14.4	5.1 × 10^-4^	15.0

IOSCs are based on ZnO nanostructures grown in a solution with different Al concentrations from 0 to 4 mM.

For the convenience of comparison, the length of the AZO NFs is kept at approximately 100 nm. Clearly, devices with un-doped ZnO have a bad performance: *J*_SC _= 8.56 mA cm^-2^, open-circuit voltage (*V*_OC_) = 0.360 V, FF = 0.337, and a PCE of 1.04%. With doping 1 and 2 mM Al concentrations, the *J*_SC _of devices increases to 10.26 and 11.08 mA cm^-2^, and the PCE increases to 1.26% and 1.44%, respectively. With increasing Al concentrations to 4 mM, the *J*_SC _has hardly any change at all; however, the FF increases 0.412, and as a result, the highest PCE of 1.65% is achieved. The results indicate that the PCE of the devices can be easily improved through a simple Al doping into ZnO lattice process.

We reckon that the PCE improvement in the IOSCs is almost attributed to the high *σ *of the flake-like AZO nanostructures. Previous works had reported the *σ *of un-doped and Al-doped ZnO whiskers synthesized via hydrothermal method. The conductivities of un-doped and Al-doped ZnO whiskers were calculated using the following equation:

(1) $$\frac{1}{\sigma }=R\frac{S}{\begin{array}{c} h \end{array}},$$

where *σ *was the electrical conductivity, *R *was the tested value of resistivity, *S *was the area of the effective measuring electrode, and *h *was the thickness of the samples. It is found that the *σ *of AZO whisker samples significantly increased with the increase of Al concentrations [16]. In this study, the value of *σ *is calculated using the same method, as shown in Table 1. It is found that the *σ *increases dramatically from 8.2 × 10^-5 ^to 5.1 × 10^-4 ^(Ω cm)^-1 ^with the increase of the Al concentrations from 0 to 4 mM. Compared with the un-doped ZnO nanostructures, the AZO nanostructures can provide a higher conduction pathway to enhance electron transport from P3HT/PCBM matrix to FTO electrodes, as can be clearly clarified in the dark current of devices in Figure 3c. The AZO-based devices have a lower leakage current and *R*_S _than that of the un-doped ZnO-based devices. The results are shown in Table 1. Therefore, through increasing the *σ *of the AZO to facilitate electron transport, the *J*_SC _and PCE of IOSCs can be strikingly improved.

In addition, we turn to consider that a suitable morphology of AZO NF array, such as the density of AZO NF arrays, could account for the performance increase in the IOSCs. As is well known, the lowest unoccupied molecular orbital (LUMO) level of ZnO is -4.2 eV and the LUMO level of P3HT is -3.0 eV [17]. It suggests that electrons can be injected from the LUMO of P3HT into the LUMO of ZnO. In this study, upon the Al-doping treatment, it is interesting to observe that the ultrathin and disperse AZO NF arrays have grown on the densely packed ZnO NR array. Generally, the ultrathin and disperse AZO NFs could reach the matrix of P3HT/PCBM, providing a continued "tentacles" for electron collection from PCBM clusters to FTO electrodes. At the same time, owing to the achievement of higher density of AZO NFs, the larger area of interface between AZO NFs and P3HT crystals may induce more sufficient exciton dissociation. The above two factors could explain why the *J*_SC _of IOSCs would drop slightly as increasing Al concentration from 2 to 4 mM, even if the *σ *continues to increase.

To better understand the role of the AZO morphology on the performance of the IOSCs and eliminate the possible effect of different PCBM cluster aggregations on the IOSC performance simultaneously, we fabricated two hybrid solar cells A and B with the structure of FTO/ZnO seed layer/AZO nanostructures/P3HT/Al. The AZO nanostructures, synthesized in a solution of 2 and 4 mM Al concentrations respectively, have a significant variety in morphology, such as the density and the size. The *J*-*V *characteristics of the IOSC devices are presented in Figure 3d. It has been found that the *σ *increases with the increase of Al concentrations from 2 to 4 mM; however, the *J*_SC _decreases from 2.73 to 2.37 mA cm^-2 ^and the FF increases from 0.311 to 0.320, and as a result, the PCE decreases from 0.22% to 0.19%. It suggests that less interfacial area between the P3HT and the AZO nanostructures induces a less exciton dissociation, even though AZO nanostructures of a higher *σ *have been achieved through more Al doping into ZnO lattices. Therefore, a sufficient interface contact for exciton dissociation between P3HT and AZO nanostructures should account for the increase in the PCE of IOSCs.

## Conclusions

In conclusion, we fabricated the IOSCs based on the flake-like AZO nanostructures with controllable *σ *and morphology via a simple hydrothermal method. The effects of the electrical property and morphology characteristics of flake-like AZO nanostructures on the performance of the IOSC devices were investigated. It is found that the PCE of the IOSC devices depends critically on the *σ *of AZO nanostructures and the interface area between the flake-like AZO nanostructures and the active layer. These results suggest that the flake-like AZO nanostructures could be a promising potential candidate in various photovoltaic and optoelectronic applications to improve device performance.

## Competing interests

The authors declare that they have no competing interests.

## Authors' contributions

All authors contributed equally and read and approved the final manuscript.
